# Prevalence and Practice of Unused and Expired Medicine—A Community-Based Study among Saudi Adults in Riyadh, Saudi Arabia

**DOI:** 10.1155/2020/6539251

**Published:** 2020-07-06

**Authors:** Syed Wajid, Nasir A. Siddiqui, Ramzi A. Mothana, Sana Samreen

**Affiliations:** ^1^Department of Clinical Pharmacy, College of Pharmacy, King Saud University, Riyadh, Saudi Arabia; ^2^Department of Pharmacognosy, College of Pharmacy, King Saud University, Riyadh, Saudi Arabia; ^3^Department of Pharmacy, College of Pharmacy, Aurobindo College of Pharmaceutical Sciences, Warangal, India

## Abstract

**Purpose:**

The objective of the current study was to determine the prevalence and practice of unused and expired medicine among Saudi adults. *Subjects and Methods*. The study used cross-sectional web-based design to collect the data, over a period of 4 months among people who are living in Riyadh, Saudi Arabia.

**Results:**

A total of 337 questionnaires were obtained during the study period. The majority of respondents were university graduates *n* = 251 (74.7%). The prevalence of unused medicine was *n* = 301 (89.3%). The most commonly used drugs were nonsteroidal anti-inflammatory drugs *n* = 272 (80.7%) and antibiotics *n* = 164 (48.7%). Of the participants, 186 (55.2%) checked the expiry date of the medicine before they purchase. Most of them *n* = 305 (90.5%) obtained medicine through prescription. About *n* = 219 (65%) of them keep the medicine until it expired; 48.1% throw away in the household garbage while only 18 (5.4%) of the respondents said to give it back to the medical store.

**Conclusion:**

The study revealed a high prevalence of unused medications among Saudi community. However, the disposable practice among the Saudi community was inadequate. Increasing awareness through education programs about proper disposable guidelines is necessary for controlling the medication wastage.

## 1. Introduction

Pharmaceuticals remained a top priority for the government bodies in both national and international levels, contributing to a significant rise in the overall healthcare cost. Medicines are substances used for the diagnosis, treatment, or disease prevention and for controlling the symptoms associated with the disease, as well as for cosmetic reasons and for lifestyle behavior modification [1–3].The use of drugs and medicinal substances has to gain popularity for keeping one's health perfectly. A large number of drugs and nutraceuticals available in the market with or without prescription, additionally overprescribing, were also found by a number of studies around the world including Saudi Arabia [4–8]. These contribute to the accumulation of excess quantity of medicine, in homes as well as in the environment, which results in toxic effects on the wildlife [9].

It is estimated that the economic cost of medication wastage was $150 million annually among Saudi families [10], while the mean medication wastage was 25.8% in Saudi Arabia and 41.3% in other gulf countries [4, 10]. This not only is an essential problem in Saudi Arabia but also contributes universally in both developed and developing countries [10]. It is also evidenced that the accumulation of unused or expired medicine leads to a significant for diversion, misuse, and abuse which may even lead to overdose [11].

There were a number of guidelines and regulations published by many developed countries such as the United States [12], United Kingdom [13], and Australian [14] government for the appropriate disposable practice and for controlling the deposits of leftover medicines in homes through take-back policy, the return of medicines by patients to pharmaceutical stores or manufacturer, and the collection of unwanted medications by inspectors who visit homes [15, 16]. However, in Saudi Arabia, literature shows that there is no standard established policy to control medication waste management. Additionally, in Saudi Arabia, citizens were provided by free healthcare cost with access to medicine by the ministry of health through various health channels. Furthermore, the literature suggests that a lack of awareness and knowledge about drug use and adherence to medications might increase the number of unused medicine in homes [17–20]. Therefore, it is essential to assess the prevalence and trends in unused and expired medicine among the Saudi public. The present study was undertaken to evaluate the prevalence and trends towards the disposal of leftover medicine in the Saudi community.

## 2. Methods

### 2.1. Study Design, Settings, and Sample Size Estimation

A cross-sectional web-based study was carried out over a period of 4 months from March 2019 to June 2019 using a simple random sampling technique, in which subjects were randomly invited to participate in the study. The study included adults who were able to read and understand Arabic language, both genders aged 18 years and above, and Saudis who were living in the capital of Saudi Arabia. According to the previous prevalence rate of unused medications in Saudi homes (25.8%) [21], the sample size (*N*) was calculated using the following formula: *N* = *z*^2^ × *p* × *q*/*d*^2^, where *N* is the minimum sample size, *z* is the level of confidence according the normal standard distribution which corresponds to the 95% confidence interval (*z* = 1.96), *p* is the prevalence of unused medications (0.258), *q* = (1 − *p*), and *d* is the desired degree of accuracy or tolerated margin of error which is 5% (0.05).

(1) $$\begin{array}{c} N={\left(1.96\right)}^{2}\times 0.26\times \left(1-0.26\right)/\left(0.05\right)2=295,\\ N=295\text{ people}. \end{array}$$

### 2.2. Study Questionnaire and Data Collection

An anonymous research survey was prepared to estimate the prevalence and practice of leftover medicine among Saudi adults in Saudi Arabia. A self-administered validated questionnaire was adapted from the previous studies published in this regard [19, 20, 22]. The study questionnaire included demographic details of the respondents like age, sex, marital status, educational level, and a question about place of or region of living in Saudi Arabia. The participants were asked questions about ways of purchasing medicines, classes of medicines used, whether they checked the expiry date before purchasing a medicine, and whether any quantity of medicines remains unused at their home. We also asked the participants of what they did with unused and expired medicines and those who are responsible for creating awareness about the proper disposal of such medicines. The questionnaire contained 11 items with the options to fill in the blank and multiple-choice questions.

### 2.3. Questionnaire Validation and Administration

A pilot study was conducted before proceeding for the original study with a sample of 10 randomly selected participants. The pilot study was to ensure that the target population understood what each research question was asking, as well as what each response meant. A research team consisting of a researcher and two professors (one from college of nursing and another from college of pharmacy) were involved and discussed the ideas in the pilot study on how to make the survey easier to the subjects. According to the results of the pilot study, the research questionnaire were revised. The results of the pilot study were not included in the research. Reliability of the questionnaire was assessed with the Cronbach's alpha coefficient of 0.7. All Saudi adults were enrolled through the random sampling technique using different social media. An invitation link containing a survey questionnaire was sent to the participants randomly without any previous measures. For the data collection, we used the snowball technique where any person recruited to do the survey provides multiple referrals.

#### 2.3.1. Data Analysis

The data was extracted to exclude the bias in the sample selection which was limited to only the central region and analyzed by using Microsoft Excel worksheet, and then, descriptive analysis was performed. Categorical data was calculated as frequencies and percentages. Statistical Package for Social Sciences version 22.0 (SPSS Inc., Chicago, IL, USA) was used for statistical analysis.

## 3. Results

The questionnaire was filled by 337 individuals with a male predominance shown by 83% of the population. The demographic characteristics of respondents in the study sample are shown in Table 1. Among the participants, 59.5% were single and 40.7% were married. The majority of respondents were university graduates (74.7%).

**Figure 1:**
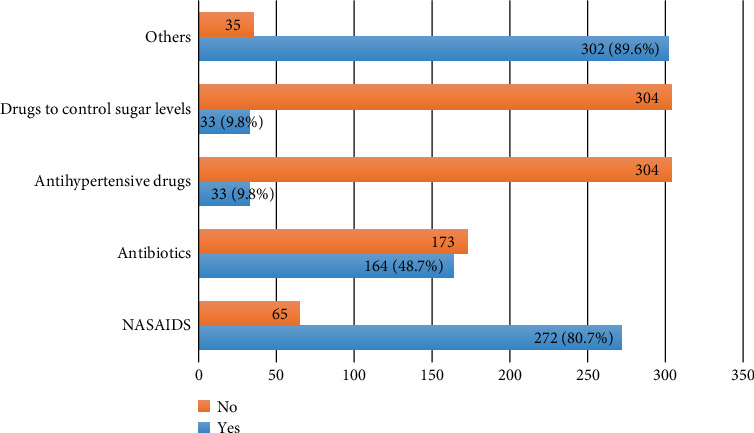
The most common classes of drugs used by participants.

The prevalence of unused or expired medicine among Saudi community was 89.3%. The most commonly used drugs among the participants are shown in Figure 1. The majority (80.9%) of respondents said that they used miscellaneous drugs (which includes inhalers, sprays, asthmatic drugs, and cosmetics) while 80.7% used nonsteroidal anti-inflammatory drugs, although some of the respondents used antibiotics.

Regarding knowledge about checking the expiry date, more than half 55.2% of the interviewed participants checked the expiry date of the medicine before they purchase, while 5.6% of them do not know about it. When we asked about the methods of obtaining medications, majority, 305 (90.5%), of respondents obtained through prescription, more than half 181 (53.7%) obtained without a prescription (OTC), and 61 (18.2%) brought it upon the advice of a friend or relatives. (Table 2).

When we asked about what you do with unused or expired medicine, 219 (65%) of the respondents keep the medicine until it expired while 48.1% of them throw away in the household garbage, and 46 (13.7%) of them agreed to give it to friends or relatives; only 18 (5.4%) of the respondents said they give it back to the medical store. Additionally, a good majority of the respondents, 288 (85.5%), said that the government is responsible for creating awareness about the proper disposal of unused and expired medicines while 129 (38.3%) of them agreed that it should be the pharmaceutical industries. Almost all of the respondents, 311 (92.3%), accepted that improper disposal of unused and expired medicines affects the environment and health. (Table 2).

## 4. Discussion

In the context of drugs and pharmaceuticals in both developed and developing countries, pharmaceutical industries are urged to provide a greater contribution to health budgets in the healthcare system [18]. This research demonstrates the prevalence and practice of unused medications and may assist in providing importance and awareness about the disposable practice of unused medicines, among the Saudi public to minimize medication wastage, although the present study found relatively high prevalence of unused medicine among the Saudi community, which is similar to previous results [9, 19–22]. Additionally, most of the respondents kept unused medicine until it expired. However, a large number of participants know that improper disposable of pharmaceuticals causes undesirable effects on the environment as well as on living beings.

In our study, majority of the respondents obtained medicines in the prescriptions; similar results were found in Kabul [19], while another study by Braund R et al. in 2009 using an online survey, reported that 56% of the participants purchased medicine from the doctor's prescription; also, the author reported a high prevalence of unused medicine among the New Zealand population [9]. However, a number of studies also reported that the presence of leftover medicine is due to multiple reasons such as the medical condition improved with different treatment, simply an excess quantity of supply, expired medicine, or fear of side effects [9].

The present study results are also similar to the other studies in terms of types of medicine present [19–22]. Our study reported that most commonly leftover medicines were painkillers, analgesics, and antibiotics, and small proportions were for diabetics and hypertensives. These results were comparable to the study by Mohammad Bashaar and Koshok MI [19–23].

United States Food Drug Authority (USFDA) had launched several guidelines for the safe disposal of unused and expired medicine which includes the take-back program and household trash flushing the medicine. However, in Saudi Arabia, our results show that 48.1% of participants throw unused medicine in the household garbage which is similar to the study done in the USA and Ethiopia [20, 24] where 54% of them use household trash to dispose unwanted medicine. Similarly in the UK, 63% of participants also used household garbage for disposing leftover medicine [25]. However, our study results were lower than the study done by Mohammad Bashaar et al. where 77.7% of the participants discarded the expired medicines in household trash [19].

In Nigeria, a recent study assessed the disposable practice among community pharmacies; results found that 31.8% of the participants followed the National Agency for Food and Drug Administration and Control guidelines for the disposable of unwanted medicine (NAFDAC); 23.9% gave back to drug distributors, and 9.1% of cps used rubbish [26]. In our study, only 5.4% of Saudi participants flushed the unwanted medicine in the toilets, and only 5.4% of them returned unused medicine to medical stores. In the New Zealand study, participants indicated that between 13 and 24% of medications are returned to a pharmacy [9]. These results suggested that in Saudi Arabia, still no adequate and standard guidelines were established for the safe and proper disposal of unused medicine.

Environmental pollution is the major cause of improper disposable of medication wastage as reported by many studies [27, 28]. The present study also reported similar results and shows that the majority of the Saudi participants agreed that the Ministry of Health, pharmaceutical industries, and pharmacist were responsible for creating awareness among the public for safe disposable practice [19, 20]. Additionally, a large number of Saudi participants were aware that improper disposable leads to destruction and damage to health environment which is similar to studies done in both developed and developing countries [19, 20].

The results of the present study evidenced that in Saudi Arabia, clear guidance about the disposal of unused and expired medicines is lacking, and there is a deficit in practicing appropriate methods for medicines disposal [28]; also, results suggested that most of the participants were practicing irrational methods of disposing medications. Previous studies reported that lack of awareness might be the reason for wrong disposable practice [28]. It is potentially recommended to establish national-level drug disposable practice in the Saudi government which is vital for the safety of the environment and for controlling the medication wastage by individuals.

However, this study has some limitations. Firstly, the study included only Saudi nationals and is limited to the capital of Saudi Arabia. The generalizability of our findings should thus be evaluated in future studies. Additionally, we recommend the importance of assessment of the prevalence of leftover medicine using noninvasive tools like questionnaires. This type of assessment may help the policymakers to assess the problems of the society timely and make necessary recommendations.

## 5. Conclusion

In conclusion, the study revealed that a high prevalence of unused medications among The Saudi community was observed. However, the practice of disposable was moderate in the Saudi community. Additionally the majority of the Saudi participants were aware of the toxic effects of medicine on the environment. Increasing awareness through education programs about proper disposable guidelines is necessary for controlling the medication wastage. Future studies are needed to explore this important topic of medication wastage and how to tackle it.

## Figures and Tables

**Table 1:** demographics of characteristics of the study subjects (*N* = 337).

Participant classifications	*N*	%
Gender		
Male	280	83.1
Female	57	16.9
Marital status		
Single	200	59.5
Married	137	40.7
Level of education		
Primary	3	0.8
Secondary	45	13.4
University	251	74.7
Postgraduation	38	11.3
Monthly income		
Excellent	53	15.8
Very good	102	30.3
Good	106	31.5
Average	46	13.7
Weak	29	8.6
Did any quantity of the medicine purchased remain unused in your home?		
Yes	301	89.3
No	36	10.7

**Table 2:** Respondents' practices concerning unused and expired medication.

Variables	*N*	%
Do you check the expiry date of the medicine before procuring?		
Yes	186	55.2
No	132	39.2
Do not know	19	5.6
Ways of purchasing medicines		
Purchased using a prescription	305	90.5
Purchased without prescriptions (OTC)	181	53.7
Received from friends/colleagues	33	9.8
Brought/purchase upon the advice of a relative	61	18.2
What do you do with unused medicines or expired medicine?		
Throw away in the household garbage	162	48.1
Donate to a hospital	13	3.9
Give to friends or relatives	46	13.7
Return to medical stores	18	5.4
Keep at home until expiry	219	65
Flush unused medicines in the toilet	18	5.4
Who is responsible for creating awareness about the proper disposal of unused and expired medicines?		
Government	288	85.5
Pharmaceutical industries	129	38.3
Public	60	17.9
Pharmacist	152	45.2
Can improper disposal of unused and expired medicines affect the environment and health?		
Yes	311	92.3
No	26	7.7

## Data Availability

The datasets generated and analyzed are available from the corresponding author in a reasonable request.
